# Learning to Control Actions: Transfer Effects following a Procedural Cognitive Control Computerized Training

**DOI:** 10.1371/journal.pone.0119992

**Published:** 2015-03-23

**Authors:** Nitzan Shahar, Nachshon Meiran

**Affiliations:** Department of Psychology and Zlotowski Center for Neuroscience, Ben-Gurion University of the Negev, Beer-Sheva, Israel; University of Tokyo, JAPAN

## Abstract

Few studies have addressed action control training. In the current study, participants were trained over 19 days in an adaptive training task that demanded constant switching, maintenance and updating of novel action rules. Participants completed an executive functions battery before and after training that estimated processing speed, working memory updating, set-shifting, response inhibition and fluid intelligence. Participants in the training group showed greater improvement than a no-contact control group in processing speed, indicated by reduced reaction times in speeded classification tasks. No other systematic group differences were found across the different pre-post measurements. Ex-Gaussian fitting of the reaction-time distribution revealed that the reaction time reduction observed among trained participants was restricted to the right tail of the distribution, previously shown to be related to working memory. Furthermore, training effects were only found in classification tasks that required participants to maintain novel stimulus-response rules in mind, supporting the notion that the training improved working memory abilities. Training benefits were maintained in a 10-month follow-up, indicating relatively long-lasting effects. The authors conclude that training improved action-related working memory abilities.

## Introduction

"Never mistake motion for action" (Ernest Hemingway).

The ability to control, monitor and execute actions in a goal-directed manner has been the focus of much research [1–4]. Usually, our environment holds multiple action cues, most of which are irrelevant to the internal plan being pursued. Additionally, in everyday life, there is a frequent need to shift between one or more tasks. To move between task-sets in a flexible, goal-directed manner, the cognitive system needs to update and maintain relevant action information in mind while resisting interference from irrelevant environmental cues. Thus, following internal action plans might require the orchestrated operation of distinct control process such as inhibition, shifting and updating of action rules. The ability to exert control over actions was found to have a broad relevance to major issues such as cognitive development [5,6], aging [7,8], attention- deficit/hyperactivity disorder [9–11], depression [12–14], frontal lobe damage [15] and intelligence [16–18]. Thus, finding ways to improve these control processes would potentially have a significant impact. The aim of the current study was to explore a novel training protocol, explicitly targeting action control process.

Although most studies refer to cognitive control functions (or executive functions) as one set of control process, it is unclear whether *procedural* control processes (which monitor and regulate actions) are distinct from *declarative* processes (relevant to knowledge and facts). Studies that address executive functions typically use the taxonomy of three control processes: updating of information in working memory, inhibition of a pre-potent response and switching between tasks or mental sets [1]. In this taxonomy, there is no distinction between procedural and declarative sub-systems. Yet, paradigms that tap response inhibition [19,20] or switching [21–23] are usually based on measurements of procedural control (e.g., stopping an already initiated response or switching tasks). In contrast, paradigms that tap working memory updating abilities tend to do so using (almost exclusively) declarative representations. For example, a common working memory updating paradigm is the N-back task [24–26]. In the N-back task, participants are asked to indicate whether a current target stimulus is identical to the target stimulus presented N trials back. Therefore, in this task participants are required to hold and update *declarative* representations in working memory as procedural demands (remembering the task rules) remain low and do not increase with N. Complex span tests [26–28] are also widely used to measure working memory abilities. In these tests, participants are asked to remember information while performing a distracting task. Although these tasks might be procedurally demanding (switching between a memorizing task and the distraction task), the primary dependent measure is the amount of information successfully remembered, i.e., the declarative aspect. Additionally, the load is manipulated by increasing the number of items that need to be remembered, thus not changing the procedural demands of the task.

Recently, Oberauer [29] suggested a distinction between procedural and declarative working memory, with declarative working memory being responsible for the maintenance of facts and knowledge and procedural working memory being responsible for maintaining representations needed for the execution of the current task. This hypothesis has gained empirical support [30,31]. Specifically, Souza, et al., [30] manipulated declarative and procedural working memory load in a single task and found an under-additive interaction. This result led the authors to conclude that the updating and maintenance of representations in working memory is processed in two distinct sub-systems, procedural and declarative. Notably, there is evidence that the ability to execute complex and novel task rules might be more predictive of fluid intelligence than declarative-based working memory tasks [16,32]. This finding gives some additional support to the distinction between procedural and declarative working memory sub-systems.

In the last decade, much interest has been devoted to the study of cognitive training, exploring the mechanisms that might allow the improvement of cognitive processes using computerized training tasks. Currently, there is a wealth of training studies focusing on working memory updating training, predominantly relying on tasks that tax declarative working memory [33–36]. By contrast, only a limited number of studies have directly examined the trainability of the action control process, and none of the training studies of which we are aware used exceptionally demanding training tasks in terms of procedural working memory [37].

The few studies that did explore the effects of action control training made use of the task-switching paradigm as a training task. The task-switching paradigm [21,23,38–41] has been used extensively to explore action control processes, specifically the ability to organized and follow complex task rules. In this paradigm, participants are asked to switch between two or more tasks, with a cost in performance being observed in trials that demand a task switch compared to trials in which no switching is required (i.e., switch cost). It is important to note that the task switching paradigm is assumed to be demanding not only in terms of switching but also in terms of procedural working memory updating. For example, Mayr and Kliegl [42] argued that task switching requires the retrieval of action rules (i.e., stimulus-response rules) of the upcoming task into working memory. Thus, an important aspect of task switching includes the ability to maintain, update and shield the task rules in working memory [43]. The task switching paradigm is also considered demanding in terms of inhibition. First, each target stimulus in this paradigm conveys information about multiple tasks. Thus, if, for example, the participant is asked to perform Task A, the cognitive system might need to refrain from responding according to information relevant to Task B that is also conveyed by the target stimulus. When the responses conveyed by the target for Task A and Task B mismatch, a cost is observed (i.e., task rule incongruence effect) [44]. Additionally, switching has been claimed to require the ability to inhibit the previous task [45].

One leading example of a study in which the task switching paradigm served for training is Karbach & Kray’s [46]. These authors gave three age groups (i.e., children, young adults and older adults) either task-switching training or a control training task across four sessions. Both the training and the control groups performed a two-choice reaction task, requiring participants to make simple perceptual judgments (e.g., big/small, plane/train, etc.). The control group performed each of these tasks separately, in single blocks, and the training group performed mixed blocks, demanding task switching. The authors found beneficial transfer effects in similar switching tasks, with the training group demonstrating better switching abilities. In addition, the results demonstrated a far transfer effect to measurements of working memory, inhibition and even fluid intelligence. In a second study that examined a similar training only with children suffering attention-deficit/hyperactivity disorder, the authors found similar improvements, though with no transfer to fluid intelligence measurements [47]. Yet, the utility of this training protocol has been questioned, particularly given the difficulty to replicate the far transfer effects originally reported by Karbach and Kray [48,49].

Additional evidence for training action control process comes from action video game studies [50]. These studies use commercial action video games as a training task. The games require the player to learn and react according to a complex set of rules and procedures. Thus, action video games can be a real-life approximation of the action control process demanded in laboratory paradigms. Several correlational studies have found that experienced video game players demonstrate lower switching costs than novice players [51–53].

Given the correlational nature of these studies, one cannot rule out the possibility that people who choose to play video games have better switching ability. This interpretation is ruled out given that similar evidence has been found using experimental designs. Strobach et al. [54] examined novice video game participants in a pre-post battery of executive function tasks (i.e., dual task and task switching). Three groups were included in this study: an experimental group, an active control group and a no-contact control group. Participants in the experimental group were trained for 15 hours in an action control-demanding video game (i.e., Medal of Honor). The training game required constant monitoring and switching between multiple game-related actions and was performed under strong time constraints. The active control group was trained for the same amount of hours in a computerized puzzle game (i.e., Tetris). The puzzle game was also demanding in terms of executive function (i.e., mental rotation) but required focusing on only one task. Last, a no-contact comparison control group performed only the pre- and post-test measurements. The results demonstrated improved performance in task switching, reflected by the reduced switch costs for the experimental group compared to both the active and no-contact control groups. Similar improvement in action control was found in the dual-task paradigm. Others have found similar evidence for improvement in the action control process following training in action video games. For example, Green et al. trained novice video game participants in an action video game for approximately 50 hours and found a greater reduction in switch costs following action video games training compared with control training [55].

To conclude, research using task switching training is characterized by fixed (and moderate) levels of task demands. Moreover, although near transfer effects seem replicable, far transfer effects are more difficult to replicate. Video game playing seems more promising in this regard, yet the involvement of executive functions in the video game is rather implicit given that these are commercial games that were not explicitly designed to tap specific aspects of action control.

In this study, we designed a novel training protocol that explicitly targeted critical components of action control. We used a task with an adaptive difficulty level and a high training dosage (i.e., 19 training sessions), based on results from previous studies [56,57]. The training task required participants to randomly switch between two choice-reaction tasks. In each trial, a cue appeared, signaling which task should be performed, followed by the target stimulus. In addition, when the difficulty level increased, the participants were asked to react according to the stimulus (either the cue or the target) that appeared N trials beforehand. For example, participants could have been presented with a cue for Task A but had to perform Task B because this was the required task N trials beforehand. Thus, unlike the N-back training previously used [56], the current N-back element was mostly action-related; it demanded that the participants maintain and update the representation related to the task rules and not merely be able to report a piece of information. The task difficulty was adjusted according to participants’ performance using multiple sets of variables (see Method section), including the N value. Thus, the training task remained very demanding throughout the training. In addition, to prevent as much as possible performance improvement due to the formation of long-term memory traces and to keep the working memory demands high, a new set of stimuli (i.e., task cues and target stimuli) and response keys was introduced in each block of the training task.

The training group was compared to a no-contact control group who underwent just pre-testing and post-testing. We chose to use a no-contact control group for two main reasons. First, this was the first study to test this particular training protocol, and as such the study was regarded as preliminary. Second and not less importantly, a recent meta-analysis of working-memory training in young adults [58] indicated that although control-group type (active vs. no-contact) influenced the Experimental-vs.-Control difference in pretest-to-posttest gain, this control-group type effect was exclusively due to the experimental groups—that is, it was independent of the type of the control group that was studied. Other studies have also demonstrated no substantial difference between passive and active control groups [59]. Thus, we chose a no-contact comparison group that controls for pre-testing effects [60,61]. To be on the safe side, we were extra cautious in hiding group membership information (see below), to prevent any biases due to demand characteristics and other types of expectations.

We predicted that practice would increase pretest-to-posttest in tasks that require executive control (i.e., near transfer). Additionally, we also explored the improvement in fluid intelligence following training (i.e., far transfer). We chose to include measurements of fluid intelligence based on previous reports [35,56,58,59,62] and on Duncan et al. [16,32], who showed that the ability to maintain and execute complex and novel task rules might be more predictive of fluid intelligence than are declarative based tasks. To examine such near and far transfer effects, a battery of executive functions was administered before and after 19 training sessions. The measurement battery was designed to estimate processing speed, switching, response inhibition, and working memory updating (i.e., near transfer), as well as fluid intelligence measures (i.e., far transfer). In addition, participants completed a follow-up measurement session administrated 10 months from the end of training, testing for long-term transfer effects. Aside from testing the stability of the gains, the follow-up session was introduced in order to rule out some alternative accounts of the results of the first posttest.

## Method

### Participants

31 healthy undergraduate students from Ben-Gurion University of The Negev took part in the study (mean age = 25.1, SD = 1.6, 25 females) in return for either course credit, 25 NIS per hour (~$6) or an equivalent combination of both. Participants were pre-screened for self-reported head injury, psychiatric disorders, drug/alcohol use, color blindness, diagnosed attention disorders and learning disabilities. The study received approval from the Ben-Gurion University psychology department’s ethics committee, subordinate to the Ben-Gurion University institutional review board (IRB). All participants provided written consent to participate in this study, using a consent form that was approved by the ethics committee.

### Procedure

The design included two groups, a training group and no-contact control group. That is, both groups underwent pre, post and follow-up measurements simultaneously, but only the training group participated in 19 training sessions in between the pre and post measurements. To keep participants and experimenters blind to the study conditions, we used a "triple-blind" procedure in which we divided the training and measurement sessions into what appeared to be as two different experiments. This design aspect allowed us to keep the participants blind to the fact that they were being tested for training related improvements. In addition, both the participants and the experimenter who ran the measurement sessions were blind to the group assignment. In the debriefing that took place after the experiment ended, none of the participants noted being aware that the measurement and training session were actually related.

After the initial screening and drop-out (see Appendix A for a full description of the recruitment process), 31 participants were randomly assigned to the training and control groups. Participants in the control group were invited to a round of training sessions that were dated after the actual study had ended (without the participants being aware of this fact). Thus, participants in the control group were similar to the training group in their intention to take part in a training study. The training group completed 19 lab sessions of 60 minutes each, over a period of 25 days. All participants completed two pre-test and two post-test sessions. After finishing the entire procedure, participants were paid and were offered a full debriefing.

### Apparatus & setting

Across the study (i.e., pre-post measurements and training sessions), participants were seated in front of a computer screen in a small room in the lab. Tasks were programmed using E-Prime 2.0 (Psychology Software Tools, Pittsburgh, PA). Stimuli were presented on a black 19" computer screen. Participants responded using a QWERTY keyboard or vocal response box according to the specific instructions for each task.

### Training Task

**Fig 1:**
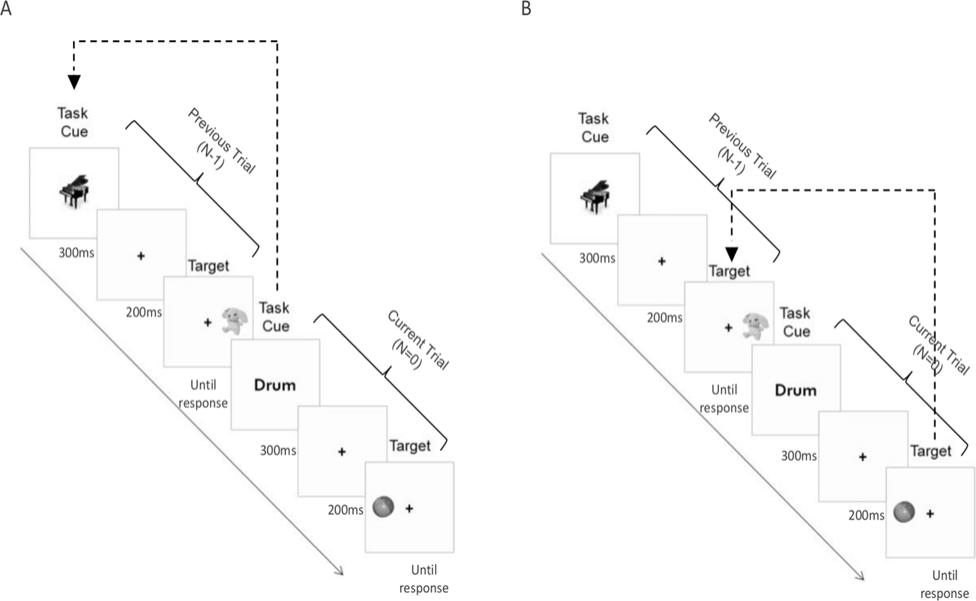
A demonstration of two trials in the training task, each trial including the presentation of a task cue (indicating which task to perform), fixation and a target stimulus. The current figure demonstrates a condition of N = 1, requiring participants to respond according to the task cue (panel A) or the target (panel B) presented in the previous trial (i.e., N = 1)

Participants were asked to switch between two 2-alternative choice reaction tasks: an object classification task (e.g., report using a key press whether a plant or flower is presented on a computer screen) and a spatial classification task (e.g., indicate, using a key press, whether the image is displayed on the right or left side of the screen; see Fig. 1). Sessions 1 to 4 included ten blocks, and Sessions 5–19 included seven blocks. (The number of blocks was adjusted to ensure the task was not too long when participants made it to higher levels.) Each training block included: (1) an instruction screen presenting a novel set of stimuli and stimulus-response mapping for the spatial and object classification tasks, (2) two practice blocks (6 trials each), one for the object classification task and one for the spatial classification task, and (3) a test block (64 trials) in which the two tasks were randomly switched. Each trial sequence included a task cue (300 ms), a fixation point (200 ms) and the target stimulus (presented until a response or until 6000 ms had elapsed). A "chimes" (500 ms) and a "beep" sound signaled correct and error responses, respectively. Across the training blocks, the following aspects were manipulated:

#### N-back

Participants were asked to respond according to the stimuli presented N trials beforehand. Whether the N-back aspect referred to the target stimulus or the cue stimulus was randomly selected at the beginning of each training block. When N-back referred to the target stimulus, participants had to execute the task instructed by the current task cue. When it referred to the task cue, they had to respond to the current target stimulus. The N value in the current block was set according to the participants' performance in the previous block (see Table A in S1 File, Table B in S1 File, and Fig. 1).

#### Stimuli & response keys

To increase task novelty, each training block included a randomly selected new set of task cues, target stimuli and responses. There were 16 sets of object stimuli (i.e., plant/flower, boat/plane, etc.), 7 sets of locations (i.e., up/right, down/left, near/far, etc.), 4 sets of arbitrary task cues (i.e., dog/cat, piano/drum, etc.) and 7 sets of keyboard responses (i.e., S/K, T/B, etc.), resulting in as many as 6,272 possible combinations for each training block (see Table A in S1 File).

#### Task cue modality

The task cue modality also varied randomly between trials so that the task cue could appear in one of three modalities (i.e., text, image, sound; see Table A & B in S1 File).

#### Response threshold

A different reaction time (RT) criterion was selected randomly in each training block, demanding participants to generate response times that would be either below or above 800 ms (see Table A in S1 File).

#### Task rule incongruence

To prevent participants from employing a constant control policy, the proportion of task rule incongruent trials was manipulated between blocks. The effect of rule congruency is the poorer performance seen when the target stimulus invokes a competing response according to the task that is not currently required. The proportion of task rule incongruent trials was adjusted each level (see Table A & B in S1 File for details).

#### Mapping compatibility

The compatibility of the stimulus-response rules in the spatial task with the response key position also varied between blocks. Three levels of mapping compatibility were employed: (1) compatible mapping (e.g., "right" reported using a key positioned on the right side of the QWERTY keyboard), (2) neutral (e.g., "right" reported using a key positioned on the upper side of the QWERTY keyboard) and (3) incompatible (e.g., "right" reported using a key positioned on the left side of the QWERTY keyboard). Mapping compatibility (i.e., compatible, neutral or incompatible) was adjusted according to the participant level (see Table A & B in S1 File for details).

#### Fadeout trials

When participants are required to perform a single task in a block (i.e., no task switching was required) immediately after performing a mixed block (where switching was required), the first trials tended to show prolonged RTs, reflecting the difficulty of reducing control, even when the instructions specifically indicated that no alternation between tasks would be demanded [13,63]. Thus, to prevent participants from adhering to a particular control mode (related to switching, for example), participants were asked twice during each test block to perform only one task (i.e., either spatial or object task, randomly selected), with N = 0, and to respond very quickly (RT<500 ms). This demand appeared in a randomly chosen trial during the test block, and it lasted 8 trials. These fadeout phases were indicated by a thick, black border surrounding the target stimulus.

#### Adaptive difficulty and level adjustment

Task difficulty was determined according to the participants' accuracy rate in the previous block. If the participant had an accuracy rate above 80%, the difficulty level was incremented by one step. If performance accuracy dropped below 60%, the level was lowered by one step. That is, the difficulty was changed based on the total number of errors in the previous block. In each level, the following parameters were adjusted: the task cue dimension, the proportion of task incongruent trials, and mapping compatibility (see Table B in S1 File). Additionally, N-Level was incremented by one each fourth level (i.e., N = 0 for levels 1–4, N = 1 for levels 5–8, N = 2 for levels 9–12, N = 3 for levels 13–16, and N = 4 for levels 17–20; see Table B in S1 File). The lagged increase in N-level was chosen to make the difficulty adjustment more gradual. This was important given the difficult nature of this training task, even under low N-Levels.

### Pre-post measurements

The pre-post measurement battery was designed to estimate five domains of executive function: processing speed [64–66], switching [21–23], response inhibition [19,20], working memory updating [29] and fluid intelligence [67,68].

#### Digits updating task

Performance in the digit updating task was used to estimate *working memory updating* abilities. The working memory updating task was similar to that used by Kessler & Meiran [69]. In this task, the participants were asked to memorize as quickly as possible three digits presented on the screen inside three square frames. After memorizing, the participants were asked to press the space bar, the frames went blank, and a simple arithmetic operation (addition or subtraction) appeared inside one of the three frames. The participants were then asked to overtly perform the arithmetic operation on the last number that was related to the frame and to press the space bar as quickly as possible when done. The task included 10 blocks, each including six updating trials, at the end of which the participants were asked to recall the outcome digits related to each of the three frames.

#### Shape classification task

Performance in the shape classification task was used to estimate *switching* and *processing speed*. In each trial, a shape (diamond or triangle) was displayed with a different filling (i.e., full or empty). Participants were asked to perform one of the two choice tasks, shape (diamond vs. triangle) categorization or (2) filling (empty vs. full) task. In both tasks, the same response keys were used (i.e., S (left), K (right), in a QWERTY keyboard). Participants performed six blocks in the following order: two single-task blocks (one for each task), two mixed-tasks blocks (where the two tasks switched randomly) and, finally, two additional single-task blocks. The tasks were ordered in a sandwich design (e.g., shape, filling, mix, mix, filling, shape). Whether the first task was the shape or filling task was counterbalanced across participants. Each of the six blocks included 48 trials. Each trial started with the presentation of a textual Hebrew task cue (i.e., “color” or “filling”; 300 ms), followed by a presentation of both the cue and the shape target (until a manual response was given or 6 seconds had elapsed). The first three blocks (i.e., two single blocks and one mixed block) also included a short practice phase, which included six trials in which the stimulus-response mapping appeared beside the target.

#### Vocal Stroop

Performance in the vocal Stroop task was used to estimate *switching* and *response inhibition* abilities. One of four Hebrew color words (i.e., the Hebrew equivalent of “green”, “blue”, “red” or “purple”) appeared in colored ink (i.e., green, blue, red or purple) at the center of the screen. Stimuli were either congruent (i.e., the word "red" in red ink) or incongruent (i.e., the word "red" in blue ink). In each trial, participants were asked to name either the written text (i.e., the word-reading task) or the color of the ink in which the word was written (i.e., the color-naming task). The task started with two single-task blocks, where each task (i.e., word-reading or color naming) was performed separately. This was followed by two mixed-blocks, where the two tasks switched randomly. Finally, two additional single-task blocks were performed. Thus, this task included four single-task blocks and two mixed blocks in a sandwich design (e.g., ink, word, mix, mix, word, ink). Whether the first task was word-reading or color-naming was counterbalanced across participants. Each of the six blocks in the task contained 48 trials (12 congruent, 36 incongruent trials). In each trial, a cue (i.e., Hebrew text indicating “color” or “word”) indicating the task appeared (100 ms), followed by a presentation of both the cue and the Stroop stimulus (presented until a vocal response was given or until 6 seconds had elapsed). Finally, a blank screen was presented (700 ms). Vocal reaction time was recorded using a homemade voice key that was attached to a microphone.

#### Stop-signal

[70] Performance in the stop-signal task was used to estimate *response inhibition* abilities. In this task, participants were asked to indicate whether the current trial included the presentation of a circle or square by pressing one of two keys (i.e., Z, / keys, left/right on a QWERTY keyboard). In each trial, a fixation was displayed for 250 ms, followed by the target stimulus (i.e., circle or a square). The target stimulus remained on the screen until a response was given or until 1,250 ms had elapsed. Furthermore, a beep sound was played at random in 25% of the trials along with the target stimulus, signaling to participants to refrain from responding. The presentation of the stop single (i.e., beep sound; 750 Hz presented for 75 ms) was delayed in some trials, according to participants’ performance, with longer delays making it more difficult to stop an already initiated response. The task consisted of a practice phase of 32 trials proceeded by a test phase of three blocks of 64 trials. This task was performed using the STOPIT software, which is freely available [70].

#### Digit classification task

Performance in the digit classification task was used to estimate *processing speed* abilities. In this task, participants were asked to indicate whether a current trial included the presentation of the digit 1, 2 or 3 by pressing the matched key on a QWERTY numbers keypad. The task consisted of two blocks of 72 trials. Each trial included a fixation point (500 ms) followed by the target stimulus presented in the middle of the screen until a response was given.

#### Fluid intelligence

Raven's Advanced Progressive Matrices (RAPM) Test was used to estimate *fluid intelligence* abilities [71,72]. To allow for two parallel forms to be used in the pre-post design, the test was divided into two forms using even and odd items (i.e., form A, form B), which resulted in 18 items in each form. To counterbalance the difficulty differences between the forms, half of the participants received form A in the pre-test and form B in the post-test and the other half in the opposite order. The tests were computerized so that a test item was presented on the screen, and participants had to key-in the response they chose. Participants were given 15 min to complete the test. The test ended with the participant either answered all 18 items or exceeded the time limit.

## Results

### Training

**Fig 2:**
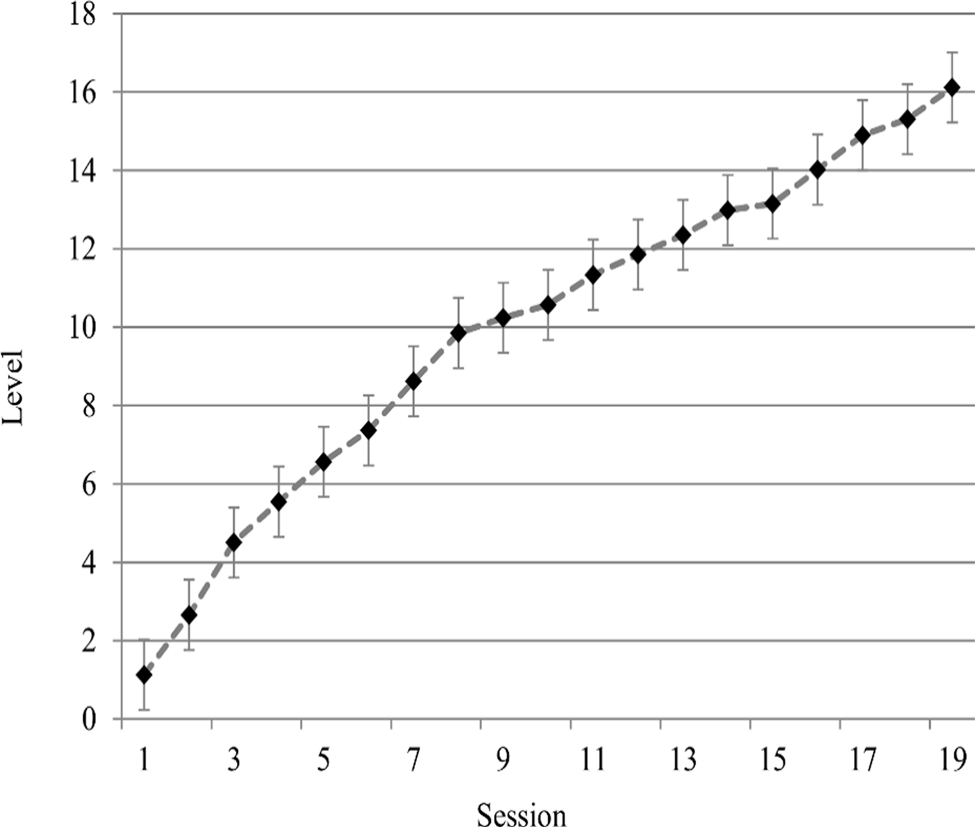
The mean level in each training session, calculated across participants. Bars represent 95% confidence intervals [79].

All participants in the training group completed 19 sessions of training over a period of 25 days. At the end of the training period, participants had completed, on average, 141.07 blocks, ranging from a minimum of 133 blocks to a maximum of 148 blocks. The mean level at the end of training was 16.11 with a mean N-Level of 3.39 (The N level was increased every fourth level; see Table B in S1 File) A repeated measures Analysis of Variance (ANOVA) that explored the effect of Session (1 to 19) on participants' mean level revealed a significant effect [F(18, 216) = 87.22, p<.001, η_p_^2^ = .88], indicating that participants did, in fact, improve in the training task as training advanced (see Fig. 2).

### Pre-post measurements

For RT analysis, error trials, post-error trials and the first trial in each block were omitted. In addition, 2% of the RT data in the upper and lower portion of the RT distribution were omitted for each participant in each condition. For each dependent variable, we formed a difference score by subtracting the post score from the pre-score. To explore the training effects, we formed five groups of measurements, arranged based on a priori theoretical consideration:

#### Switching

The estimation of switching abilities was obtained by calculating (1) switch cost: the difference between the RT to the switching trials (i.e., trials in which the previous task was a different task) and the RT to the repeat trials (i.e., trials in which the previous task was the same as the one in the current trial), both performed in the mixed-tasks blocks and (2) alternation cost: the difference in RT between the mixed tasks blocks (i.e., where the task occasionally switched) and the single-task blocks (i.e., where no task switching occurred). Each of these costs was calculated separately for the shape classification and vocal Stroop tasks. Because the switch cost in the Stroop task is known to show an asymmetric effect, we included only trials with the word-reading task to calculate switch costs. In addition, the alternation cost in the Stroop task was calculated individually for the color-naming and word-reading tasks.

#### Response Inhibition

Response inhibition was estimated using (1) the STOPIT estimated Stop-Signal RT (describing the time needed to stop a response that has already been initiated), which was calculated from the stop-signal task, and (2) the Stroop interference, which was calculated by the difference in RT between incongruent trials and congruent trials for the color-naming task. This was calculated using trials from single blocks, where no task switching was required.

#### Working memory updating

Working memory updating was estimated using the RT and accuracy rates for that task.

#### Processing speed

Processing speed was estimated using the RT and accuracy rates from the shape classification tasks (only single blocks) and the digit classification task.

#### Fluid Intelligence

Fluid intelligence was estimated using the total score in the RAPM.

### Analyzing group differences in pre-post measurements

To control for α inflation, we performed five multivariate ANOVAs (MANOVAs) on the difference scores with Group (control vs. training) as an independent variable. Each time, all the pre-to-post-test difference scores belonging to a given group of variables were entered as dependent variables. Moreover, to control for α value of the entire study, we divided the individual α values by the number of MANOVAs so that the α for an individual MANOVA was. 05/5 = .01. As seen in Table 1, group differences were found only in measurements of processing speed. Thus, we continued analyzing only measurements in that variable group. (See Table 2 for full descriptive statistics.)

**Table 1:** Group Differences in Transfer Measurements.

Measurements	Wilk's Lambda	F value	p value
Processing Speed	0.58	F_(2,28)_ = 9.98	<0.01
Switch Costs	0.87	F_(5,25)_ = 0.77	ns
Inhibition	0.93	F_(2,28)_ = 0.91	ns
Working Memory	0.85	F_(2,28)_ = 2.48	ns
Fluid Intelligence		t_(29)_ = 1.25	ns

**Table 2:** Pre-Post Performance Measurements for the Training and Control Groups.

Dependent Variable	Session	Training Group	Control Group
Processing Speed			
Shape classification task (RT, ms)	Pre	437 (52)	439 (75)
(arbitrary mapping)	Post	359 (38)	415 (61)
Digit classification task (RT, ms)	Pre	433 (35)	422 (54)
(non-arbitrary mapping)	Post	394 (41)	397 (52)
Switching			
Alternation cost (ms)	Pre	303 (98)	348 (140)
(shape classification task)	Post	199 (78)	303 (144)
Switch cost (ms)	Pre	124 (71)	178 (92)
(shape classification task)	Post	79 (72)	139 (141)
Alternation cost (ms)	Pre	479 (171)	527 (224)
(word-reading, vocal Stroop)	Post	318 (196)	415 (277)
Alternation cost (ms)	Pre	281 (104)	376 (165)
(color-naming task)	Post	231 (148)	397 (148)
Switch cost (ms)	Pre	78 (172)	129 (178)
(word-reading, vocal Stroop)	Post	30 (117)	76 (104)
Response Inhibition			
Stop-signal RT (ms)	Pre	233 (26)	235 (35)
	Post	229 (34)	229 (27)
Stroop Interference (ms)	Pre	78 (70)	68 (85)
	Post	73 (52)	102 (57)
Working memory updating			
Accuracy (proportion)	Pre	0.97 (0.05)	0.97 (0.06)
	Post	0.98 (0.04)	0.91 (0.11)
RT (ms)	Pre	2720 (984)	2778 (1,556)
	Post	2573 (1,082)	2445 (1,655)
Fluid intelligence			
Accuracy (proportion)	Pre	0.58 (0.22)	0.63 (0.18)
	Post	0.72 (0.20)	0.67 (0.12)

### Analyzing group differences in measurements of processing speed

**Fig 3:**
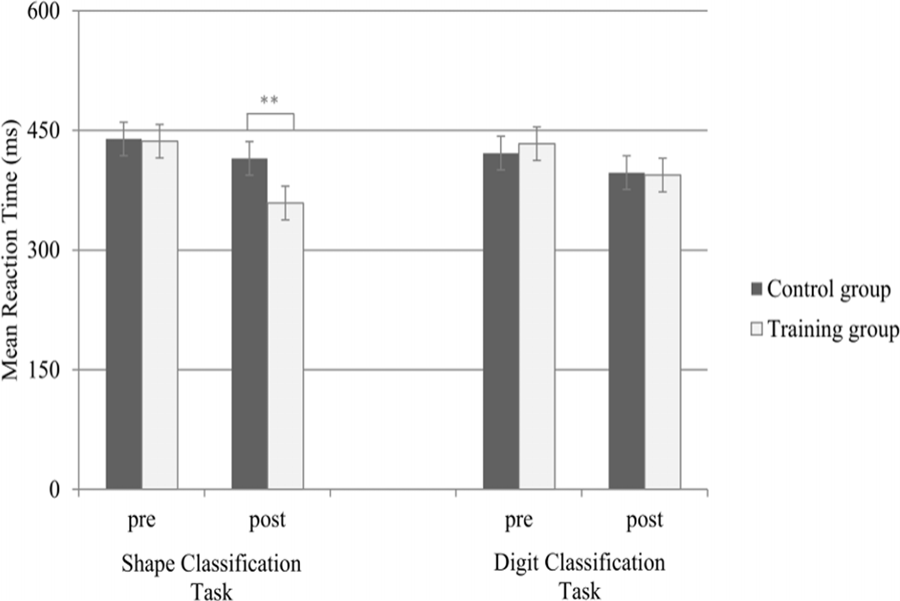
Mean RT (in ms) for the two measurement sessions (pre vs. post), the shape classification (i.e., arbitrary mapping) and the digit classification (i.e., non-arbitrary mapping) tasks. Error bars reflect 95% confidence intervals [82].

A repeated measures ANOVA was performed with mean RT as a dependent variable, Time (pre vs. post) and Task-type (i.e., shape classification vs. digit classification) as within-subjects independent variables and Group (control vs. training) as a between-subjects independent variable (see Fig. 3). Group (training vs. control) × Time (pre vs. post) interaction was found to be substantial and statistically significant [F(1, 29) = 16.81, p<0.001, η_p_^2^ = .37], showing greater RT reduction for the training group from pre-test to post-test compared to controls (see Fig. 3). Furthermore, Group × Task-type × Time interaction was found to be marginally significant [F(1, 29) = 4.08, p = .05, η_p_^2^ = .12], indicating that the group differences were most likely inconsistent across Task-type (i.e., shape classification vs. digit classification).

To further explore the differences in mean RT between the groups separately for each Task-type (i.e., shape classification vs. digit classification), a series of planned comparison was performed. First, a planned comparison exploring the Group (trained vs. control) × Time (pre vs. post) interaction only in the shape classification task revealed a significant reduction for the training group compared to the control group [F _(1, 29)_ = 17.57, p<.001, η_p_^2^ = .38] (see Fig. 3). Further comparison of Group (trained vs. control) on the shape classification task revealed no significant group differences in the pretest [t (29) = 0.12, ns, r_pb_ = .02] but a substantial and statistically significant difference at post-test [t (29) = 3.06, p<.01, r_pb_ = .49]. Hence, group differences in the shape classification task were not observed before training and are most likely the result of training. Second, the Group (trained vs. control) × Time (pre vs. post) interaction only in the digit classification task was found to be non-significant [F_(1, 29)_ = 1.4, ns, η_p_^2^ = .05], demonstrating no group differences in this task.

To account for any speed-accuracy trade-offs, we performed a repeated measures ANOVA with error rate differences (pre minus post) as a dependent variable and Group (control vs. training) as an independent variable. We found no significant differences in accuracy between the groups [t(1,29) = 0.87, ns, r_pb_ = .16] (see Table 2 for descriptive statistics).

## Discussion

In sum, we found group differences only in tasks that tapped processing speed abilities. The fact that only the shape classification task and not the digit classification task showed a transfer effect can be explained by two accounts. First, it might be that a different set size (two alternatives for the shape classification task and three alternatives for the digit classification task) reduced the transfer effect, with the two-alternative task being more similar to the training task setting. This account is in line with some studies that show that transfer effects are primarily found when the training and transfer task share the same setting [48]. Yet, the two tasks are different from one another in another important respect. Although the shape classification task used an arbitrary stimulus-response mapping (e.g., press the right key whenever a triangle appears), the digit classification task was based on familiar, long-term memory based, stimulus-response rules (e.g., press the "1" key on the numerical keypad each time this digit appears). In that sense, the shape classification task demanded that participants hold in mind (relatively) novel action rules, whereas the digit classification task allowed participants to use action rules that were familiar to them pre-experimentally. Thus, another potential conclusion is that procedural working memory has been trained.

Recently, a relationship between working memory and a specific aspect of the RT distribution in choice-reaction tasks has been found. Specifically, working memory has been shown to be related to the heaviness of the right tail of the RT distribution. Studies that explore this relationship typically use the ex-Gaussian distribution as a model to quantify the heaviness of the right tail [73,74]. The ex-Gaussian distribution consists of Gaussian and exponential distributions and is thus described by three parameters: μ & σ, the mean and standard deviation of the Gaussian component, and τ, the decay parameter of the exponential component (1/l). Thus, μ accounts for the central tendency of the RT probability distribution. For example, increasing μ will shift the RT distribution to the right without changing variability. σ accounts for the variability of the quickest RTs. Increasing σ will cause more variance in the relatively quick RTs. τ accounts for the heaviness of the right RT-distribution tail. Increasing τ will cause a higher rate of exceptionally slow RTs. Correlational studies show that τ correlates negatively with working memory abilities [75–77]. Importantly, τ was recently shown to change as a function of experimentally induced working memory load [78], with one of the major means to increase load being the arbitrariness of the stimulus-to-response mapping.

**Fig 4:**
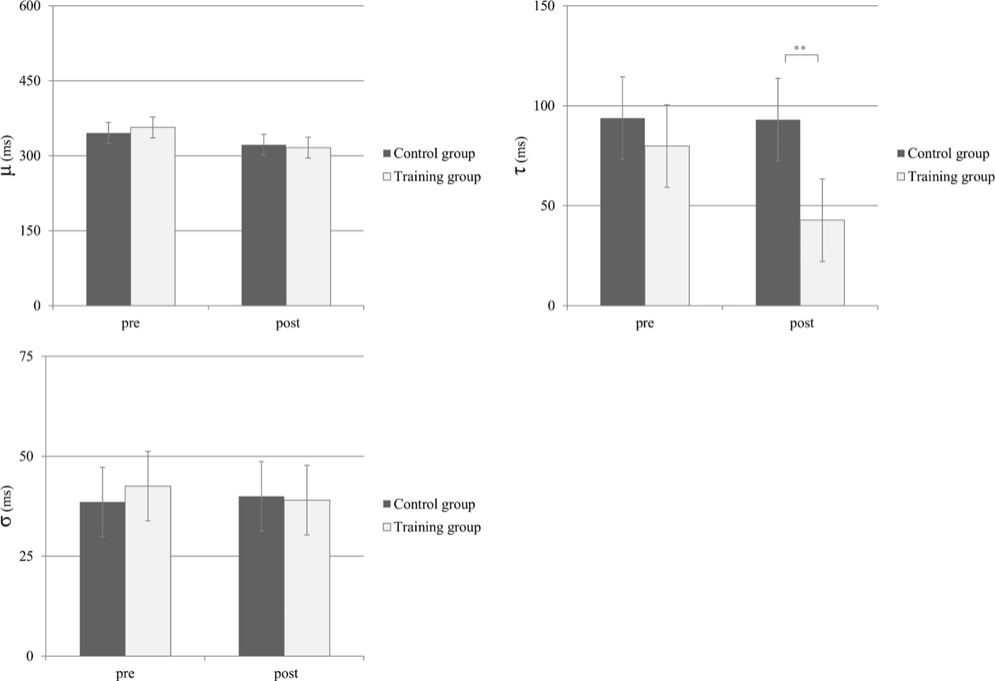
Pre-post differences for the training and control groups in each of the three ex-Gaussian parameters, estimated for the arbitrary mapping choice reaction task (i.e., shape classification). Error bars reflect 95% confidence intervals [82].

Thus, if indeed the improvement in the shape classification task was due to a better ability of maintaining arbitrary task rules in working memory, these changes should reflect in the τ parameter. To test this prediction, we performed a post-hoc ex-Gaussian distribution fitting on for the shape classification RT data (i.e., arbitrary mapping; average of 71.16 trials for each fit). Fitting was performed using the DISTRIB toolbox in MATLAB [79]. RT distributions were carefully examined visually one by one to ensure a good fit. Furthermore, a quantile-quantile plot was generated to find inconsistencies between the empirical data and theoretical ex-Gaussian distribution (see Fig. A in S1 File). After extracting ex-Gaussian parameters, we performed a separate ANOVA on each of the three ex-Gaussian parameters (μ, σ and τ) with Session (pre vs. post), Group (control, training) and Task (shapes or digits). We found a Group × Session interaction only for τ [F(1, 29) = 7.31, p<.05, η_p_^2^ = .20] and not for μ [F(1, 29) = 2.10, ns, η_p_^2^ = .07] or σ [F(1, 29) = .43, ns, η_p_^2^ = .01] (see Fig. 4). A planned comparison contrasting Group (training vs. control) in τ, only in the post-test, indicated a significant difference [t(29) = 3.52, p<.01, r_pb_ = .54]. A similar analysis on the pre-test results indicated a non-significant difference [t(29) = .91, p = .36, r_pb_ = .17].

### Follow-Up Session

The posttest results were analyzed immediately after completing the study, and this has led us to decide to run a delayed follow up session. Our considerations were as follows. First, we wanted to make sure that training effects remain after a substantial delay, especially given the fact that the specific training effects were not predicted. Additionally, the shape classification task in which we found the specific training effects involved task switching. Although the transfer effect was found only in single blocks were switching was not directly involved, these single blocks were performed in the context of a switching task. This means that although the heaviness of the right RT-distribution tail (τ) could be interpreted as reflecting the rate of rule retrieval from working-memory [78], there is an alternative explanation. Specifically, previous studies have shown that aspects of task-switching control processes selectively influence τ [80,81]. Finally, the effects were found in tasks involving shapes and we wanted to make sure this effect is not specific to this material. We therefore designed a battery of tasks that enabled us to point to the specific involvement of working-memory retrieval in the training effects.

The session included a battery of classification RT tasks that did not involve task switching (thus ruling out the involvement of switching control). The tasks were divided into three task types. (1) The critical tasks involved arbitrary stimulus-response mapping (with 2 choices, similar to the shape classification task that was used in the posttest) that forced participants to hold the mapping information in working memory. Since these were the most important tasks, we included three types of stimuli (words, shapes and numbers) to ensure that any effect is not material-specific. These critical tasks were compared with (2) classification tasks with non-arbitrary stimulus-response mapping that involved a choice but did not involve the need to retrieve stimulus-response mapping rules from working memory; and (3) a simple RT which neither involved choice nor working-memory retrieval.

Our predictions were based on the interpretation of the findings so far that training influenced working memory processing. We therefore predicted that training effects would be seen in the τ parameter of the ex-Gaussian RT distribution but only (or primarily) in the tasks with arbitrary mapping that involved working memory demand [78]. Accordingly, we did not predict any training effects in tasks with non-arbitrary stimulus-response mapping given the very little working memory involvement. Similarly, no effects were predicted in the simple RT task, which predominantly taps early perceptual and motor preparation processes.

## Method

### Participants

All participants from both the training and control groups were invited to take part in a follow-up session. 19 Nineteen participants agreed to participate (9 training, 10 controls). For their participation, participants received either a course credit or 25 NIS per hour (~$6).

### Procedure & apparatus

Participants were invited to the lab by an experimenter not known to them from either the pre-post measurement sessions or the training experiment sessions. They were told their contact information was kept in the lab and that they were contacted regarding a new experiment taking place in our lab. When asked, the experimenter explained that this experiment was unrelated to the training study. All tasks were performed in a single session and took approximately 45–60 min. Otherwise, the apparatus was the same as in the training study.

### Measurements

Three types of RT classification tasks were used: classification tasks with arbitrary mapping, classification tasks with non-arbitrary mapping and a simple RT task (without classification/choice).

#### Classification tasks with arbitrary mapping

This battery of tasks included six 2-alternatives choice reaction tasks with arbitrary stimulus-response mapping (i.e., novel mapping that was not based on any knowledge the participants might have had prior to testing). The battery included: (1) two tasks with word stimuli (classification of words describing kitchenware vs. writing tools; topics in humanities vs. topics in natural sciences), (2) two tasks with digit stimuli (classification of odd vs. even; larger than 5 vs. smaller than 5) and (3) two tasks with image stimuli (classification of ship vs. plane; skateboard vs. bicycle). Each task had a set of five stimuli per class (e.g., five images of a plane and five images of a ship). Participants were asked to classify the stimuli using the S and K keys (left and right, respectively) on a QWERTY keyboard. The stimuli were displayed using a 24-point Courier New font. Images were 6 cm × 8 cm in size. Each of the six tasks was performed for 200 trials divided into 4 blocks. Each trial began with a fixation appearing for a randomly chosen period of either 500 ms or 1000 ms. Then, the target appeared until the response was given or after 6 sec had elapsed. Error trials were signaled by a beep sound (400 ms).

#### Classification tasks with non-arbitrary mapping

In these tasks, participants were asked to perform two 2-alternatives choice reaction tasks with spatial stimuli (classification of up vs. down, left vs. right). Across the task, two borders appeared on two sides of a centered fixation marker (i.e., located above and below or on the right and left sides of the fixation marker; 7 cm × 7 cm each border). In each trial, an "X" marker (i.e., 18-point Courier New font) appeared at a randomly chosen location inside one of the two borders, and participants were required to indicate whether this was the upper/lower or right/left border. Participants responded using the Y (upper) and B (lower) keys or the S and K (left/right) keys on a QWERTY keyboard in the respective up/down or right/left classification tasks. The number of trials and trial sequence were identical to the classification choice-reaction tasks with arbitrary mapping, presented in the previous paragraph.

#### Simple RT task

Participants were asked to place the index finger of their dominant hand on the space bar and press the bar as soon as a white square (3 cm × 3 cm in size) appeared at the center of the screen. Because this task included only one possible response, no error sound was presented. The number of trials and trial sequence were identical to the classification choice-reaction tasks with arbitrary mapping, presented previously.

## Results and Discussion

**Fig 5:**
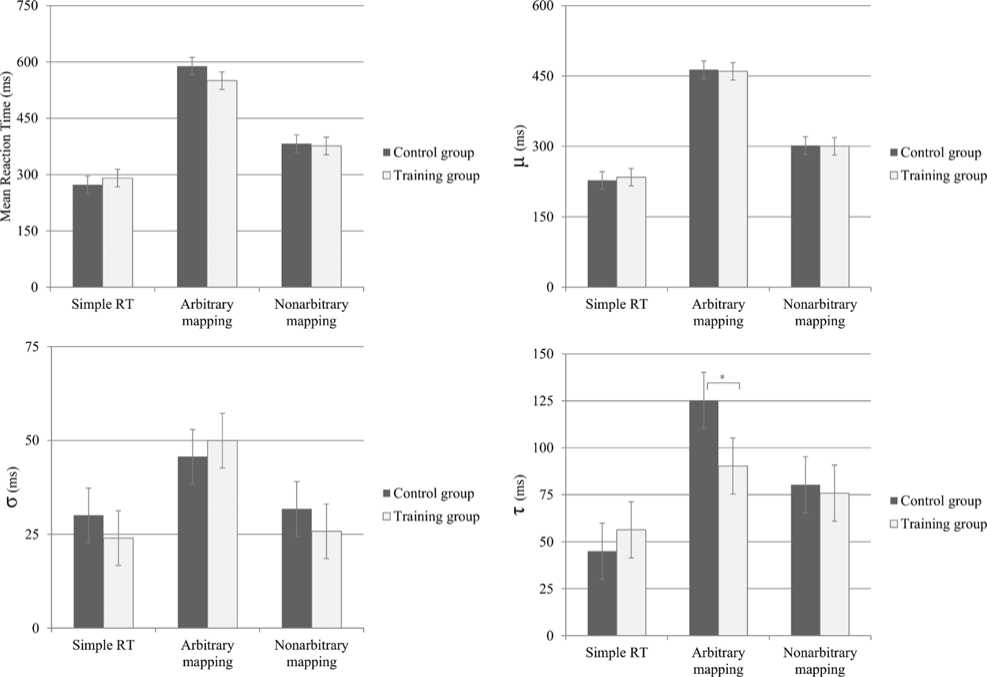
Group differences (training vs. control) in RT-means and the three ex-Gaussian parameters for each of the three tasks types performed in the follow-up session: simple reaction time and 2-alternative choice reaction with arbitrary mapping or with non-arbitrary mapping. Results demonstrate that group differences were found in the arbitrary mapping condition and only in the τ parameter, which is in line with the hypothesis that these differences are related to working memory abilities. Error bars reflect confidence intervals [82].

As we did before for RT data calculation, we omitted error trials, post-error trials, and the first trial in each block and trimmed the data by 2% in the upper and lower portion of the distribution. This resulted in an acceptable number of trials for ex-Gaussian fitting (181.47 trials on average for the simple RT task, 159.7 trials for the arbitrary mapping tasks and 171.39 trials on average for the non-arbitrary mapping tasks). We performed an ex-Gaussian distribution fitting, as we did before. After extracting the ex-Gaussian parameters for each participant in each task, we averaged the mean and each of the three parameters for the arbitrary mapping tasks (i.e., verbal × 2, visual × 2, and numerical × 2) and the non-arbitrary tasks (i.e., spatial × 2). We then performed an ANOVA on these values with Task (simple RT, non-arbitrary mapping and arbitrary mapping) and Group (training vs. control) as independent variables. This analysis was done separately for each of the four RT dependent variables: mean, μ, σ and τ (see Fig. 5). The results were in line with our predictions. We did not find any significant main effect of Group [Fs<0.76]. The interaction of Group × Task was significant for τ [F(2,34) = 4.98, p<.05, η_p_^2^ = .23] but not for μ [F(2,34) = .31, ns, η_p_^2^ = .02] or σ [F(2,34) = 1.02, ns, η_p_^2^ = .06]. One-tailed planned comparisons contrasting Group (training vs. control) with the τ estimates was found significant for arbitrary mapping [t_(17)_ = 2.31, p<.05, r_pb_ = .49] but not for simple RT [t_(17)_ = 1.05, ns, r_pb_ = .24] or non-arbitrary mapping [t_(17)_ = .29, ns, r_pb_ = .10].

The examination of error rates showed that the speed-accuracy tradeoff cannot explain the pattern of RT results because the (non-significant) pattern was similar to that seen in RT. Specifically, the mean error rates in the tasks with arbitrary stimulus-response mapping were training = .04 and control = .05. In the tasks with non-arbitrary mapping, they were. 07 and. 09, respectively.

## General Discussion

In the present study, participants were trained in a highly demanding action control task across 19 sessions. The training task required participants to (1) acquire a new set of action rules (i.e., stimulus-response rules) in each block (up to 10 times each session), (2) to constantly hold and update task rules in working memory (i.e., reacting according to the stimuli in the N-back trial) and (3) to adapt to rapid changes (i.e., switching tasks between trials). Participants in the training group were compared to a no-contact control group in a pre-post testing battery that included measurements of set-shifting, response inhibition, working memory updating, processing speed and fluid intelligence.

Participants in the training group improved only in processing speed, and there were no other systematic group differences across the different pre-post measurements. The benefits of processing speed were found only in tasks that required the maintenance of novel stimulus-response rules in working memory (i.e., arbitrary mapping) and not when those rules were familiar and/or practiced (i.e., non-arbitrary mapping), and they were restricted to the right tail of the RT distribution, as indexed by the τ parameter of the ex-Gaussian distribution, which has been linked to working memory demand [78]. A follow-up measurement demonstrated that these benefits were maintained ten months after training: Participants in the training group demonstrated similar advantages as in the post-test, in spite of the fact that the stimuli and mapping used in the follow-up study were completely new to them. Importantly, the follow-up findings enabled us to implicate working-memory retrieval processes in the training gain and also to substantially rule out the material-specificity of the effect and any involvement of task-switching.

Previous studies found that low τ values in choice reaction tasks are related to better working memory abilities [75–77] and lower working memory demands [78]. Thus, both the fact that the group differences were found only under working memory demanding conditions (i.e., arbitrary mapping) and that those changes were only found in the τ parameter support the claim that a reduction in RTs reflects better working memory abilities in the training group.

The fact that training gains were not observed in tasks with non-arbitrary mapping can also rule out the possibility that the transfer effects were due to the use of 2-choice tasks. Had this been true, training effects should have been observed in all choice tasks, regardless of mapping arbitrariness. Although the results may be interpreted as reflecting improvement in working memory, there is another viable explanation, i.e., that participants in the training group formed highly abstract representations of the stimulus-response rules, which allowed them to flexibly acquire and implement novel stimulus-response rules when needed.

Of course, there is no indication of what exactly in the training task allowed the improvement in how working memory was employed to control performance. Under conditions of a combined task-switching and N-back task, the maintenance and retrieval of action rules from procedural working memory were most demanding. Nonetheless, other action control processes, such as switching and response inhibition, were also highly demanded through the training task. The question of why only procedural working memory was improved should be further explored.

In conclusion, the present findings show that highly demanding and relatively lengthy working memory training, which emphasized action control, resulted in what appears to be an improvement in the working memory aspects of choice RT. This pattern of findings also lends some support to the claimed distinction between procedural and declarative working memory sub-systems [29–31]. An obvious caveat of the specific design is the use of a no-contact control group (rather than an active control group). The no-contact control format was chosen due to the exploratory nature of this study; this format allowed for a first examination of action control training benefits on executive functions. Future studies should thus extend the understanding of action control training on processing speed and procedural working memory and replicate the effect with an active control group.

## Supporting Information

S1 File Fig. A in S1 File, Quantile-quantile plot showing the fit between empirical data and simulated data extracted from the ex-Gaussian theoretical distributions. *Table A in S1 File*, *Variables Manipulated In the Training Task*. *Table B in S* 1 *File*, *Manipulation of the Training Task Configuration According to Training Level*.
(DOCX)
