# Supplementary material for: Learning to Control Actions: Transfer Effects following a Procedural Cognitive Control Computerized Training
Source: PLoS One. 2015 Mar 23;10(3):e0119992. doi: 10.1371/journal.pone.0119992 (PMC4370731; doi:10.1371/journal.pone.0119992)
Supplement: S1 File — Table A in S1 File, Variables Manipulated In the Training Task. Table B in S 1 File, Manipulation of the Training Task Configuration According to Training Level. (DOCX) [file pone.0119992.s001.docx]

**Supporting Information**

**Participant recruitment.**

The study was advertised to all students studying in Ben-Gurion University of the Negev via an email sent through the university system (after proper approval). Undergraduate students were invited to take part in a cognitive training study. 70 participants replied and filled a web-based admission questioner including their contact information, initial screening information (i.e., head injury, psychiatric disorders, drug/alcohol use, color blindness or diagnosed attention disorders and learning disabilities) and an indication whether or not they want to be contacted by the lab regarding additional experiments. Of the 70 participants showing interest in the study, we excluded three who noted having diagnosed psychiatric disorder, one reporting alcohol addiction, 12 reporting regular marijuana use, one reporting learning disabilities, and one reporting head injury. We also omitted six participants with dominant left hand leaving only right-handed participants in the study (to ensure group homogeneity in an effort to increase the statistical power). Two participants did not indicate they would like to be contacted by the lab regarding additional experiments. As this was crucial for the next step of the participants’ recruitment, these participants were also taken off the list. 44 remaining participants have met the screening criteria and were included in the initial participant list. In the next step, an experimenter responsible for the measurement sessions (i.e., pre and post sessions) contacted the participants and invited them to take part in the experiment. The experimenter explained that a different four sessions experiment was taking place at the lab and that he is inviting them to take part in that experiment, knowing they indicated they would like to be contact for further lab experiments. 38 participants agreed and completed two sessions of pre-test measurement. They were then contacted by an experimenter responsible for the training sessions who invited them to take part in the training experiment itself. At this point, seven participants indicated that they are no longer willing to take part in the training experiment because of having schedule difficulties. The 31 remaining participants were randomly assigned to the experimental and control groups. The control group participants told that they would be invited to a later round of training and had no training sessions between the pre-post measurements. The training group were invited to start training and completed 19 lab sessions over a period of 25 days.

**Quantile-quantile plot examining Ex-Gaussian Fitting**

A set of 10,000 data points was sampled from the theoretical ex-Gaussian distribution for each set of ex-Gaussian parameters. The mean RT for four bins (.2 .4 .6 .8), was calculated once for the empirical and once for simulated data sets. A quantile - quantile plot (see Figure A) describes the mean RT for the relevant bin in the empirical data (i.e., x-axis) and simulated data (i.e., y-axis). The current quantile - quantile plot demonstrates a very good fit, observed a minimal discrepancy from linearity.


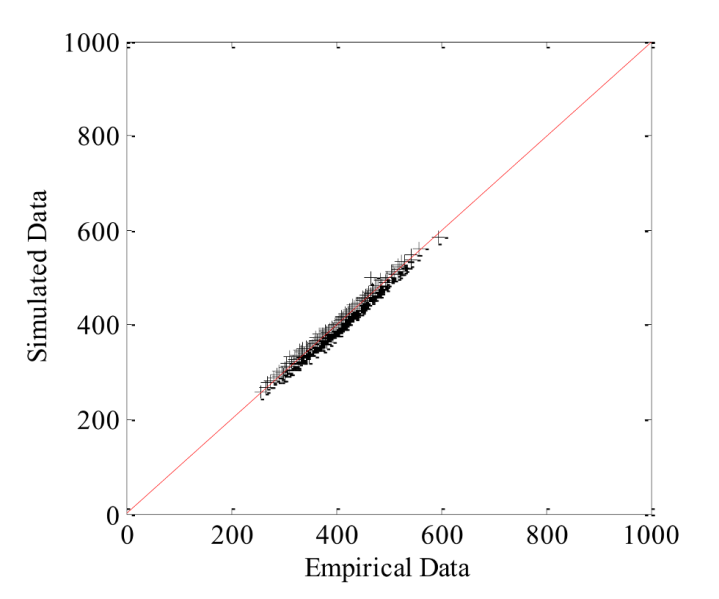


*Figure A.* Quantile-quantile plot showing the fit between empirical data and simulated data extracted from the ex-Gaussian theoretical distributions

| *Table A. Variables Manipulated In the Training Task* | | | |
| --- | --- | --- | --- |
| Variable | Manipulation Level | Selection | Dimensions |
|  |  |  |  |
| N-Back level | Block | According to training level | 0 to unlimited |
| N-Back type | Block | Randomly | According to cue / target |
| Stimuli  (targets and cues) | Block | Randomly | 10 Object sets, 12 Spatial position sets and 4 Task-cues sets |
| Response keys | Block | Randomly | 7 Sets |
| Task cue modality | Trial | Randomly and according to level | Text, sound or image. |
| Response threshold | Block | Randomly | RT<800 or RT>800 |
| Response congruency (Task rule incongruence) | Block | According to training level | 50%,60%,70% or  80% task-rule incongruent trials |
| Response congruency (Mapping compatibility) | Block | According to training level | Compatible, Neutral, Incompatible |
| Fade-out trials | Trial | Randomly | - |
| Task-type | Trial | Randomly | Object task, Spatial task |

| *Table B. Manipulation of the Training Task Configuration According to Training Level* | | | | |
| --- | --- | --- | --- | --- |
| Response congruency | |  |  |  |
| Stimulus-response compatibility | % of TRIC trials | Task cue dimensionality | N | Level |
| Compatible | 50% | Only text | 0 | 1 |
| Neutral | 60% | Text or image | 0 | 2 |
| Incompatible | 70% | Text, image or sound | 0 | 3 |
| Incompatible | 80% | Text, image or sound | 0 | 4 |
| Compatible | 50% | Only text | 1 | 5 |
| Neutral | 60% | Text or image | 1 | 6 |
| Incompatible | 70% | Text, image or sound | 1 | 7 |
| Incompatible | 80% | Text, image or sound | 1 | 8 |
| Compatible | 50% | Only text | 2 | 9 |
|  |  |  |  | … |
